# Replacing Lu-177 with Tb-161 in DOTA-TATE and PSMA-617 therapy: potential dosimetric implications for activity selection

**DOI:** 10.1186/s40658-023-00589-w

**Published:** 2023-11-10

**Authors:** Frederik A. Verburg, Erik de Blois, Stijn Koolen, Mark W. Konijnenberg

**Affiliations:** 1 Department of Radiology and Nuclear Medicine, Erasmus Medical Center, Dr Molewaterplein 40, 3015 GD Rotterdam, The Netherlands; 2 Department of Hospital Pharmacy, Erasmus MC, Rotterdam, The Netherlands; 3 Department of Medical Oncology, Erasmus MC Cancer Institute, Rotterdam, The Netherlands; 4 Department of Radiology and Nuclear Medicine, Radboud UMC, Nijmegen, The Netherlands

**Keywords:** [^177^Lu]Lu-DOTA-TATE, [^177^Lu]Lu-PSMA-617, Radionuclide therapy, Dosimetry, Terbium-161

## Abstract

**Aim:**

To explore the dosimetric effect of substituting Lu-177 with Tb-161 in targeted radionuclide therapy (TRT) using the registered tracers DOTA-TATE and PSMA-617.

**Methods:**

Using established kinetic data for [^177^Lu]Lu-DOTA-TATE and [^177^Lu]Lu-PSMA-617, radiation absorbed doses to typical tumour lesion as well as non-target tissues ([^177^Lu]Lu-DOTA-TATE: kidneys, spleen and liver, [^177^Lu]Lu-PSMA-617: kidneys, liver and salivary glands) were calculated for Lu-177 and Tb-161.

**Results:**

For both DOTA-TATE and PSMA-617, the substitution of Lu-177 with Tb-161 results in an increase in the delivered dose per unit of activity to tumour tissue by 40%. If an equivalent non-target delivered dose is strived for in order not to increase toxicity, based on kidney absorbed dose, 7400 MBq Lu-177 per cycle should be substituted with 5400 MBq Tb-161 for DOTA-TATE and 5300 MBq of Tb-161 for PSMA-617.

**Conclusion:**

When substituting Lu-177 with Tb-161, activity conversion is necessary in order not to exceed non-target dose limits.

## Introduction

Recently, the supply of the clinically important radionuclide Lutetium-177 (Lu-177) has not been secure. As the recent unplanned reactor shutdowns of November–December 2022 have shown, complete unavailability of this vital therapeutic radioisotope may occur.

A potential alternative to deal with the short supply of Lu-177 would be to replace this isotope by another, similar one. Terbium-161 (Tb-161) is a radioisotope, which has in recent years been discussed and in some instances validated [1] as a potential alternative. It is remarkably similar in both chemical properties and half-life Lu-177: 6.64 days; Tb-161: 6.95 days, as well as main beta-radiation energy Lu-177: 134 keV; Tb-161: 154 keV) and similar specific activities (Lu-177: max 4.109 PBq/g; Tb-161: max 4.356 PBq/g). However, in addition to emitted beta-radiation, decay of Tb-161 also releases conversion and Auger electrons, which may make Tb-161 a more effective isotope for use in targeted radiotherapy (TRT) [2]. We speculate that the aggregate of a slightly higher half-life, a lightly higher beta energy and the additional release of conversion and Auger electron, will significantly increase the delivered dose to both tumour and non-target tissue for Tb-161.

The aim of the present theoretical study was to explore the dosimetric effect of substituting Lu-177 with Tb-161 in TRT using the registered tracers DOTATATE and PSMA-617.

## Materials and methods

### DOTA-TATE and PSMA-617 kinetic data

For [^177^Lu]Lu-DOTATATE, we employed known historical kinetic parameters as established in the pharmacokinetic studies performed within the framework of the large phase II study performed 2001–2015 in the Erasmus MC, Rotterdam, the Netherlands [3–5].

For PSMA, we employed the kinetic and dosimetry data from a prospective pilot study on the dosimetry of [^177^Lu]Lu-PSMA-617 in 10 low-volume hormone sensitive prostate cancer patients, recently performed at the Radboud UMC, Nijmegen, the Netherlands [6, 7]. Both data sets used standard organ masses as defined in reference dosimetry phantoms. Individual patient variations have not been accounted for.

### Dosimetric modelling

**Fig. 1:**
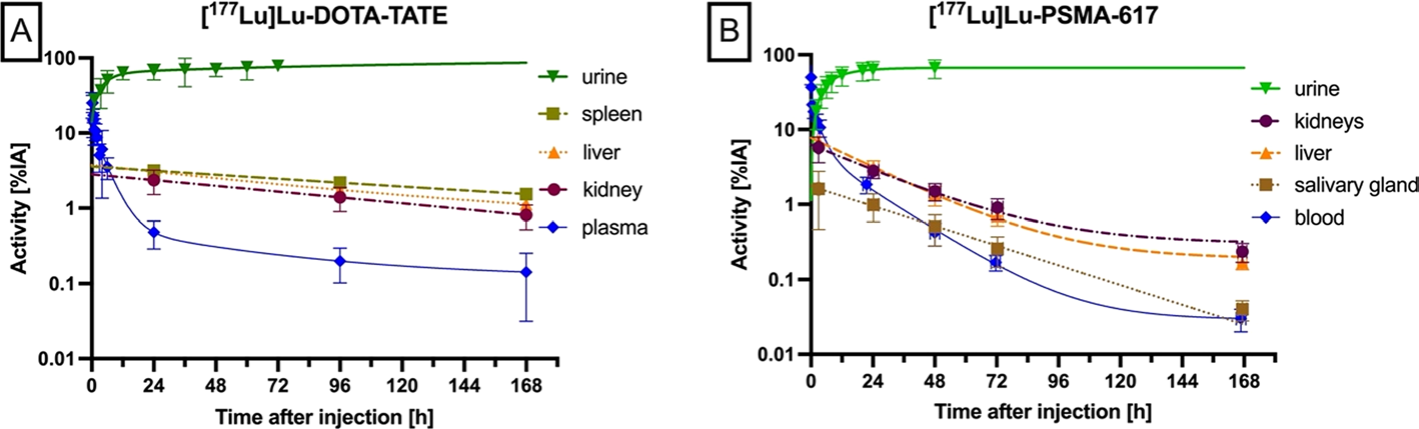
Time activity curves for urine and blood and organs after administration of **A** 7400 MBq ^177^Lu[Lu]-DOTA-TATE and **B** 6000 MBq ^177^Lu[Lu]PSMA-617. Multi-exponential curves were used to fit the urine and plasma data, single-exponential curves were fitted for all other data

The time-activity curves in organs with physiological uptake were taken from the Lu-177 clinical data (Fig. 1, Panel A). For [^177^Lu]Lu-DOTATATE physiological uptake in kidneys, spleen and liver was considered. For [^177^Lu]Lu-PSMA-617 physiologic uptake in kidneys, liver and salivary glands was taken into account (Fig. 1, Panel B). Time-integrated activity coefficients were calculated for Tb-161 and Lu-177 by integration of the exponential time-activity curves folded with the decay function of Tb-161 or Lu-177. The time-integrated activity in the urinary bladder contents was calculated with the voiding bladder model at a voiding interval of 4 h. Bone marrow uptake was modelled by the blood time-activity concentration curve assuming a red marrow to blood concentration ratio of 1. Organ absorbed doses per administered activity were calculated with the Olinda V2.2.3 (Hermes Medical solutions AB, Sweden) dosimetry software, using the adult phantoms. Tumour kinetics was estimated in PRRT by assuming 0.5% of the administered activity uptake and a biological clearance half-life of 72 h; these values were hypothetically chosen from the known broad range of values for these parameters as uptake shows a wide inter-tumour variability and biological clearance half-life shows a large inter-patient variability as detailed by our group in earlier publications [3–5]. For PSMA-617 therapy, the uptake kinetics in index lesions were taken from Peters et al. [7]; 0.1% uptake and 106 h clearance half-life. The absorbed doses to the tumours were calculated with the spheres model within Olinda V2.2.

## Results

### DOTATATE

**Fig. 2:**
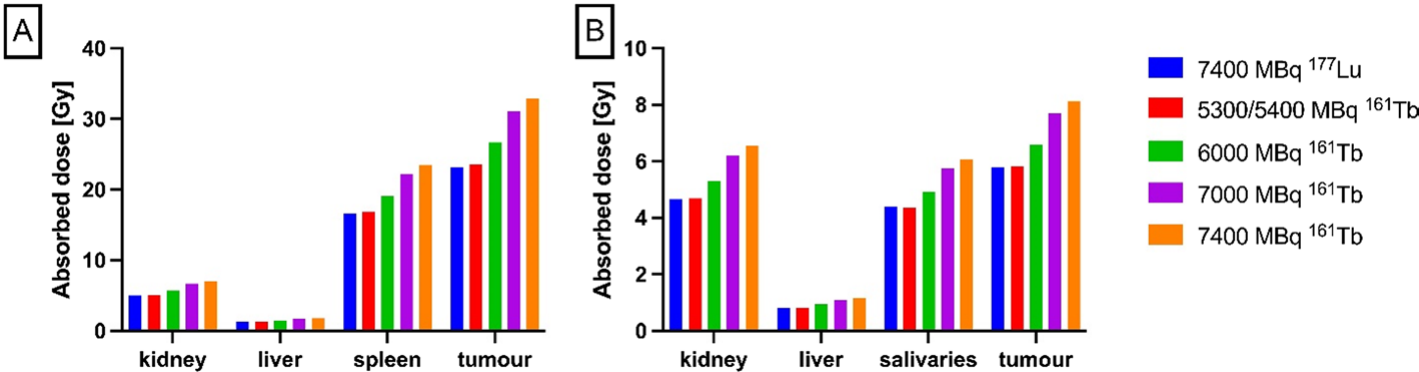
Absorbed dose to 10 g tumour and organs with physiological uptake by **A**: DOTA-TATE and **B**: PSMA-617 at 7400 MBq Lu-177 and various activities of Tb-161, starting at 5300 MBq (DOTATATE, panel **A**) / 5400 MBq (PSMA, panel **B**) as the activity with equivalent kidney absorbed dose. The examples given in this figure were chosen to represent some examples from the range between the equivalent kidney absorbed dose and the numerically identical activity

As can be seen in Table 1 and is illustrated in Fig. 2, the substitution of Lu-177 with Tb-161 results in an increase in the delivered dose per unit of activity to tumour tissue by 40% (in a 10 g tumour: 2.9 Gy/GBq and 4.1 Gy/GBq, respectively) as well as to dose-limiting non-target tissue (kidneys: 39%(0.73 Gy/GBq and 1.01 Gy/GBq, respectively), bone marrow: 42% (0.04 Gy/GBq and 0.06 Gy/GBq, respectively)). If an equivalent non-target delivered dose is strived for in order not to increase toxicity, the registered standard activity of 7400 MBq [^177^Lu]Lu-DOTATATE per therapy cycle should be replaced with a standard activity of 5400 MBq [^161^Tb]Tb-DOTATATE, based on equal kidney absorbed dose (see also Fig. 2, Panel A). The absorbed dose to the 10 g tumour would be then 22.3 Gy with 5400 MBq Tb-161, slightly higher than the 21.8 Gy by 7400 MBq Lu-177.

**Table 1:** Absorbed doses per administered activity for DOTA-TATE and PSMA-617, labelled to [^161^Tb] or to [^177^Lu, expressed in the form of the absorbed dose per unit of activity (Gy/GBq)

Absorbed dose	DOTA-TATE		PSMA-617	
[in Gy/GBq]	Tb-161	Lu-177	Tb-161	Lu-177
Kidneys	1.01	0.73	0.89	0.63
Liver	0.28	0.20	0.16	0.11
Salivary glands	0.05	0.04	0.82	0.60
Spleen	3.42	2.44	0.07	0.05
Bone marrow	0.058	0.041	0.077	0.053
10 g Tumour	4.12	2.94	1.10	0.78
1 g Tumour	40.28	28.77	10.81	7.74

#### PSMA

As can be seen in Table 1 and is illustrated in Fig. 2, the substitution of Lu-177 with Tb-161 results in an increase in the delivered dose per unit of activity to tumour tissue by 40% (0.8 Gy/GBq and 1.1 Gy/GBq, respectively, in a 10 g tumour) as well as to dose-limiting non-target tissue (salivary glands: 38% (0.60 Gy/GBq and 0.82 Gy/GBq, respectively), bone marrow: 46% (0.05 Gy/GBq and 0.08 Gy/GBq, respectively). If an equivalent non-target delivered dose is strived for in order not to increase toxicity, the registered standard activity of 7400 MBq [^177^Lu]Lu-PSMA-617 per therapy cycle should be replaced with a standard activity of 5300 MBq [^161^Tb]Tb-PSMA-617, based on equal kidney absorbed dose see also Fig. 2, Panel B).

## Discussion

The present theoretical dosimetric study provides a first comprehensive analysis of replacing Lu-177 with Tb-161 for two commonly used, registered radiopharmaceuticals. Although the differences in individual properties are small between these two isotopes, the aggregate of these differences mean that a 27–29% lower activity of Tb-161 is required to achieve the same delivered dose to target- and non-target tissue compared to Lu-177.

These theoretical considerations are roughly in line with the results reported by Baum et al., although the dose we found was slightly lower (Baum et al. reported kidney absorbed doses of 0.8 and 1.5 Gy/GBq, respectively, in two patients using [^161^Tb]Tb-DOTATOC, whereas we found 0.73 Gy/GBq using [^161^Tb]Tb-DOTATATE)—this may be caused by kinetic differences between the somatostatin receptor targeting tracers DOTATATE analysed in the present report vs. DOTATOC as used by Baum et al. [1], but more likely the larger difference with one of the two patients reported by Baum et al. was caused by the renal impairment which the particular patient was suffering from.

The major weakness of the present study concerns its theoretical nature. As Tb-161 is yet to be used in larger scale prospective studies, direct measurements of [^161^Tb]Tb-DOTATATE and [^161^Tb]Tb-PSMA-617 kinetics are as yet not available. However, considering the similar half-life of both isotopes and very similar chemical properties, there is little reason to suspect that both the biological kinetics and the rates of radiolysis will be markedly different. Although of course future prospective patient studies will have to confirm the present calculations, we nonetheless believe that the present analysis is robust enough to at least serve as a starting point for possible future prospective activity-finding phase 1 studies of [^161^Tb]Tb-DOTATATE and [^161^Tb]Tb-PSMA-617. Certainly, the agreement between the results of the same radiometal substitution between two different tracers (DOTATATE and PSMA-617) is also a strong argument to assume that the calculations here will also be valid for such similar tracers.

In theory, differences in systemic physiologic uptake between Lutetium and Terbium could cause additional differences in non-target uptake. However, ionic Lutetium and Terbium follow comparable systemic distribution patterns; uptake in skeleton and liver, as the other lanthanides [8]. Therefore, marginal difference in absorbed dose is to be expected from Tb-161 or Lu-177 coming free from the chelator.

To the best of our knowledge, Lu-177 and Tb-161 labeled tracers have not yet been compared preclinically or clinically. The principle of comparison employed in this study has been routinely used for other radiotracers, such as for the substitution of [^111^In]In-octreotide by [^177^Lu]Lu-DOTATATE [5]. The same method of substitution assuming equality of distribution was previously already published for [^177^Lu]Lu-PSMA-617 and the same tracer labelled with other radionuclides such Y-90 or Ac-225 [2]. Furthermore, in work employing the very similar tracer DOTATOC as well as the functional somatostatin receptor antagonist, Borgna et al. were able to show employing simultaneous dual-isotope SPECT that in a xenograft animal model both isotopes were interchangeable without affecting chemical and pharmacokinetic properties of the labelled tracer [9].

The present work primarily explores the activities of Lu-177 and Tb-161 which yield an equivalent non-target exposure and illustrate the effect on tumour doses based on a hypothetical example. The effect of substitution of these isotopes on a microscopic intratumoural level is beyond the scope of the present brief report but has been explored before by Bernhardt et al. [2].

A major hurdle for TRT in many countries are aspects related to radiation protection. The use of Tb-161, of which the gamma-spectrum with peaks at 43.1 keV and 74.6 keV is much more favourable in terms of radiation protection than Lu-177 with peaks at 112.9 and 208.4 keV. The lower total activity necessary to achieve the same target absorbed dose will further increase the possibility of performing TRT as an outpatient procedure.

In radiochemical terms, a lower total activity is of distinct advantage as this will improve the radiochemical stability—generally speaking, the higher the activity, the higher the rate of radiolysis.

Furthermore, a lower activity in the presently studied substitution will also result in a lower total amount of radioactive atoms required for labelling, which in turn may lead to a reduction in the total amount of precursor used. Especially in the case of the tracer DOTATATE, where clinically relevant pharmacological effects of the tracer are occasionally observed [10], a reduction of the total dose of substance used may lower the incidence of side-effects of therapy and may thus further improve the radionuclide therapy experience for the patient.

Of course, although the present results look promising for Tb-161, the supply of Tb-161 as yet is still even more fragile than Lu-177. Production and supply quality of Tb-161 needs to be scaled up, standardized and in GMP-quality.

In summary, the above results show that from a dosimetrical point of view, substitution of Lu-177 by Tb-161 is feasible, although an activity conversion needs to be applied in order not to exceed dose limits to critical non-target organs—however, this conversion may be of both radiochemical and clinical advantage. Preclinical and clinical studies to confirm these results to establish the safety and effectiveness of Tb-161 TRT are urgently needed.

## Data Availability

Data sharing is not applicable to this article as not datasets were generated or analysed during the current study.
